# Dental implant displacement complicated by epulis fissuratum and extraoral fistula in a patient with senile dementia of Alzheimer's type

**DOI:** 10.1002/ccr3.4440

**Published:** 2021-07-19

**Authors:** Damian Chybicki, Mateusz Słowik, Gaja Torbicka, Jolanta Białkowska‐Głowacka, Anna Janas‐Naze

**Affiliations:** ^1^ Department of Oral Surgery Central Clinical Hospital Medical University of Lodz Łódź Poland

**Keywords:** dentistry, dental implant, mental illness, oral surgery

## Abstract

Dental treatment of patients suffering from mental illnesses should not be neglected as good condition of oral cavity is one of the factors that determine satisfactory quality of life, not only by aesthetic but also by functional considerations.

## Graphical abstract

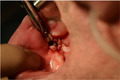

## 1 INTRODUCTION

Surgical treatment of uncooperative patients suffering from mental illnesses may be performed under short‐term general anesthesia with due care. The traditional way of excision hyperplastic tissues and extraoral fistula with the use of scalpel blade and dental implant removal by drilling bone around it is effective and does not require expensive equipment.

According to the World Health Organization (WHO) definition of dementia, it is a ‘loss of intellectual capability of such severity as to interfere with the social or occupational functioning’.^1^ Dementia is one of the main causes of dysfunction in the elderly. It is a progressive neurodegenerative disease responsible for the loss of their independent existence.^2^ Dementia can be categorized into various subtypes according to brain pathologies. The most common subtypes include Alzheimer's disease, dementia with Lewy bodies, vascular dementia, and frontotemporal dementia. Unfortunately, currently there is no known cure.^3^

Improvements in the standards of quality of life of the world's human population result in the extension of human life expectancy and an aging population. The consequence of these circumstances is an increase in the percentage of patients with senile dementia of Alzheimer's type, as age, usually above 65, is one of the risk factors for its development.^2^

Elderly people with dementia often suffer from upper respiratory tract infections and pneumonia, which may be related to poor oral health.^3^ Studies have shown that elderly people with dementia generally have poor oral health, including high risk of dental caries and periodontal disease.^4^ A study suggested that salivary gland dysfunction and poor oral hygiene are the two main reasons for unsatisfactory oral health.^3^

The effectiveness of treating senile patients with dental implants who cannot properly care for oral hygiene is controversial, due to the fact that oral hygiene is one of the most important factors in the success of this highly specialized method of treatment.^5^ The success in implant treatment is primarily due to the stable position of both the implant and the level of the bone around it, which provides predictable and satisfactory outcomes.^6^ Otherwise, the implant may become mobile or tilt buccally or lingually, or even can become completely separated from the bone. In this case report, dental implant tilted toward buccal region and caused extraoral fistula due to extensive friction.

In general, removable prostheses are commonly used in prosthetic treatment of patients with disabilities having poor oral health care, because their hygiene is fairly easy.^5^ The other treatment that combines the features of both dental implants and removable prosthesis, and improves the comfort of using a complete denture in edentulous patients is an overdenture based on implants. This kind of prosthesis was designed in the past for the described patient. The main advantage of overdenture is that it provides better retention by using implants as anchors what is important, especially in mandibular prosthesis, because the mandible creates worse prosthetic conditions than the maxilla due to the smaller support surface and the activity of the tongue.^7^

One of the many possible complications of unstable and loose prosthesis is reactive tissue response to excessive mechanical irritation, which results in the formation of epulis granuloma. Epulis granuloma is a fibrous hyperplasia, an overgrowth of intraoral tissues, which can impede the use of the prosthesis, and may also be a risk factor for cancer development due to its continuous growth due to chronic trauma and irritation.^8^, ^9^ These main factors determine that the lesion should be excised and subjected to histopathological examination.

## 2 CASE REPORT

A 81‐year‐old male incapacitated patient, suffering from senile dementia of Alzheimer's type (SDAT), was admitted to the Department of Oral Surgery, Medical University of Lodz, due to a wound on his right cheek that was noticed about 2 weeks before by his guardians. Due to the severity of his condition, the patient did not move independently and was deprived of any verbal and nonverbal contact with other people, so we were not able to collect any information about his complaints and any other details.

**FIGURE 1:**
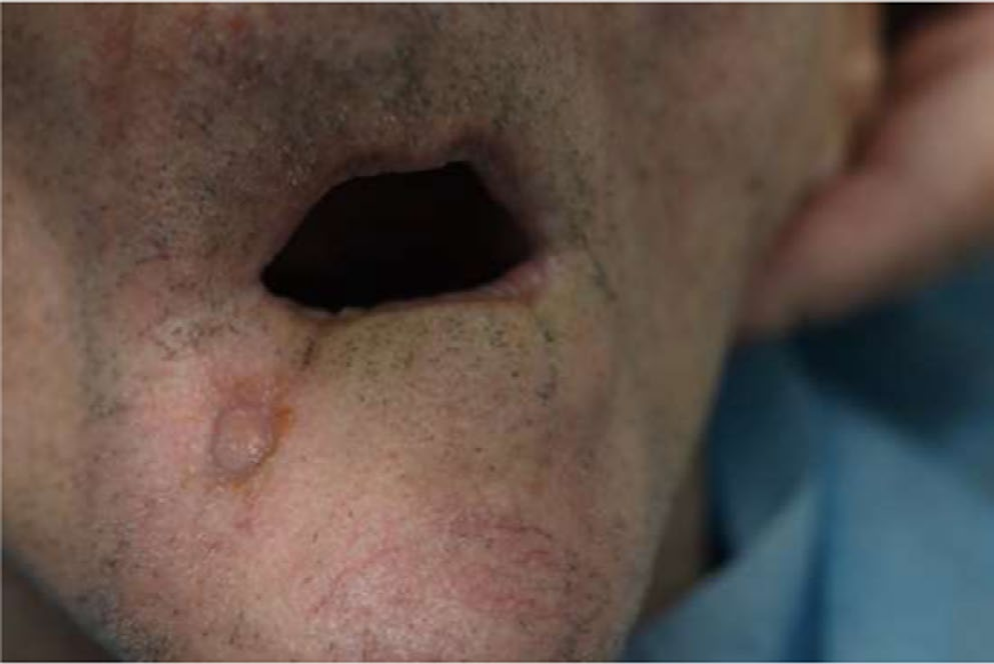
A fistula visible during extraoral examination

Extraoral examination showed fistula in the area of the right body of mandible (Figure 1), with the presence of inflammatory exudate.

**FIGURE 2:**
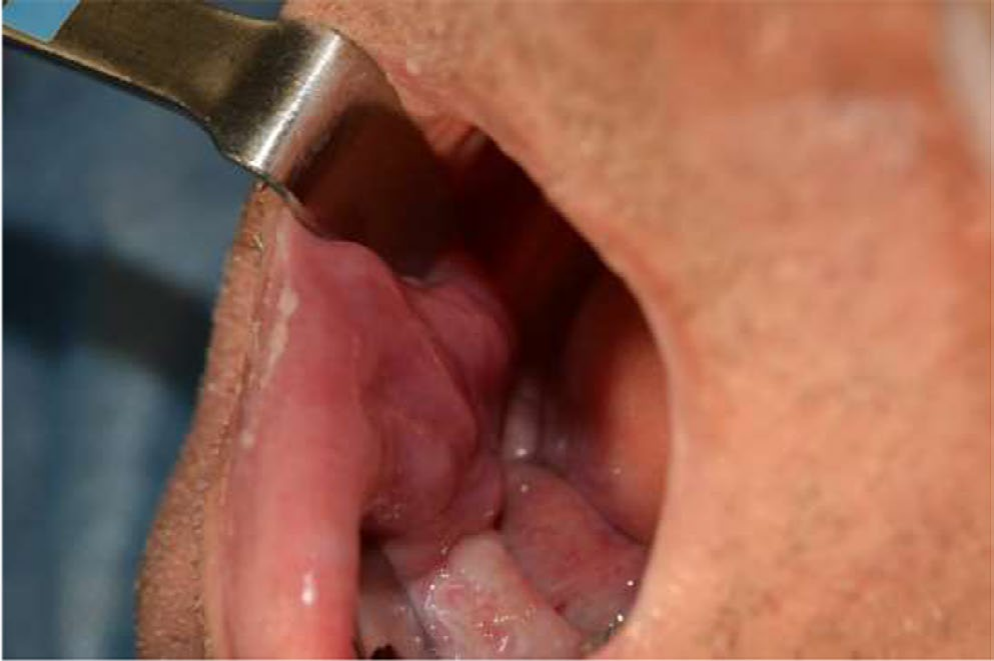
Intraoral view on operative region

In the intraoral examination, extensive hyperplasia of soft tissues was observed in the area of the right alveolar part of the mandible and vestibule of the mouth, covering the dental implant (Figure 2).

Based on clinical examination, the provisional diagnosis of implant friction fistula and epulis fissuratum was made and the treatment plan was established. The treatment plan, which included excision of hyperplastic tissues and extraoral fistula and surgical removal of dental implant, was presented to patient's guardian. The preoperative recommendations, medications, and laboratory blood tests were ordered (complete blood count, APTT), and the surgery was scheduled.

**FIGURE 3:**
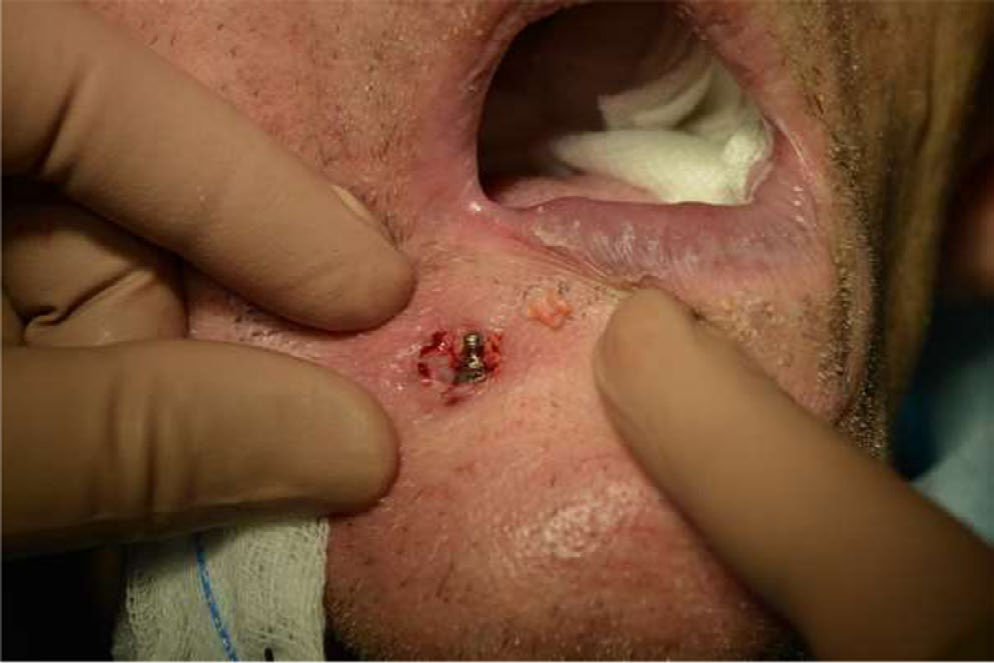
Operative region after excision of fistula, dental implant is visible through the wound

Under general anesthesia (short‐term intravenous anesthesia with the use of combination of 140 mg of propofol and 100 µg of fentanyl with monitoring the saturation and heart rate), the extraoral fistula was excised. After this procedure, dental implant becomes visible extraorally (Figure 3).

**FIGURE 4:**
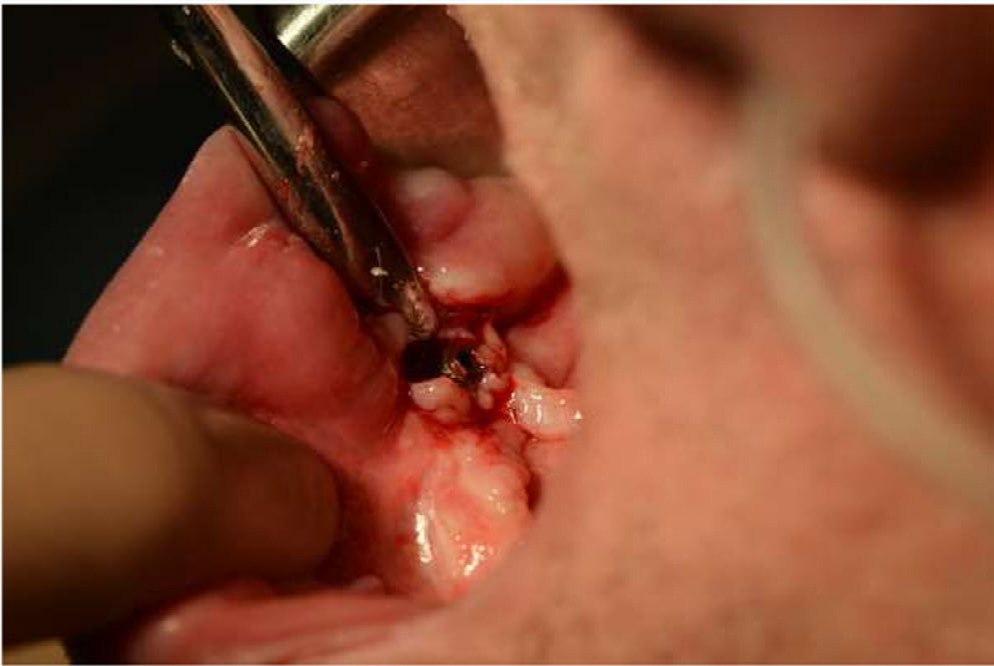
Intraoral view after incision and mucoperiosteal flap reflection

**FIGURE 5:**
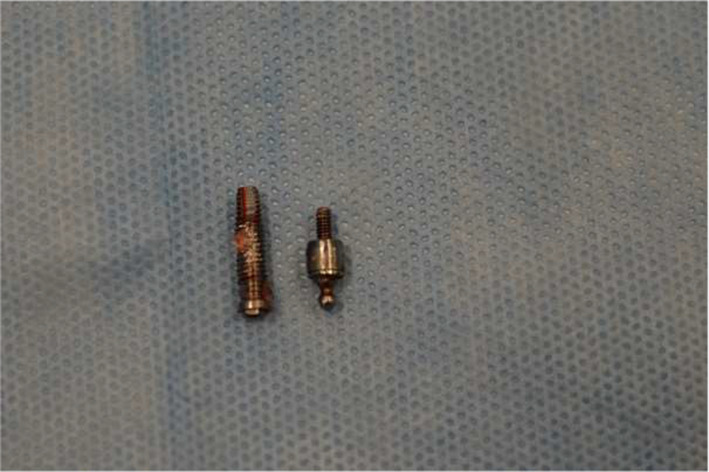
Removed dental implant

**FIGURE 6:**
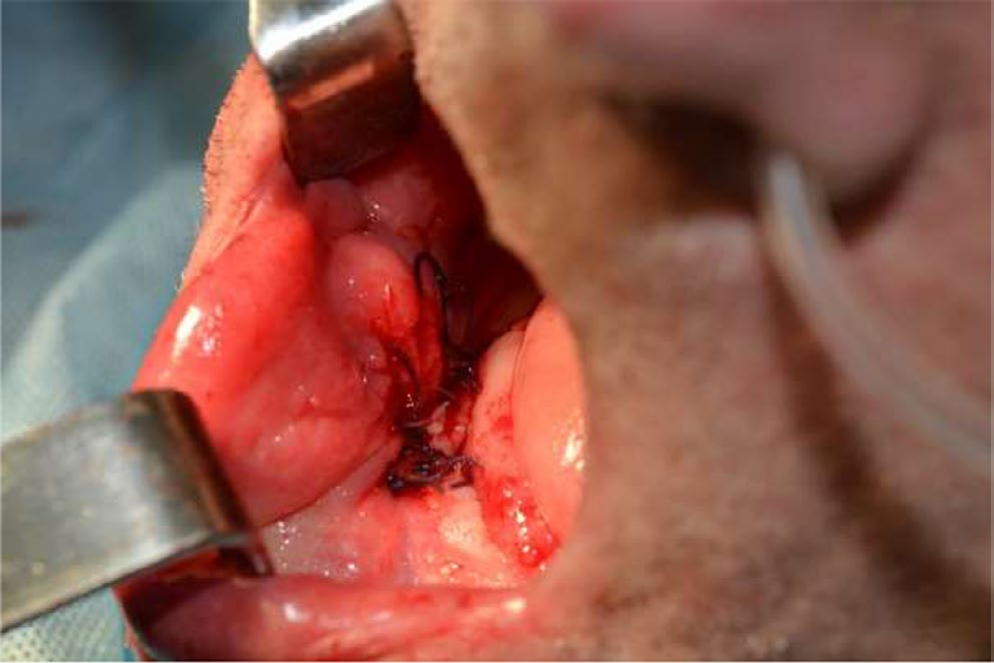
Intraoral view on operative region after suturing

**FIGURE 7:**
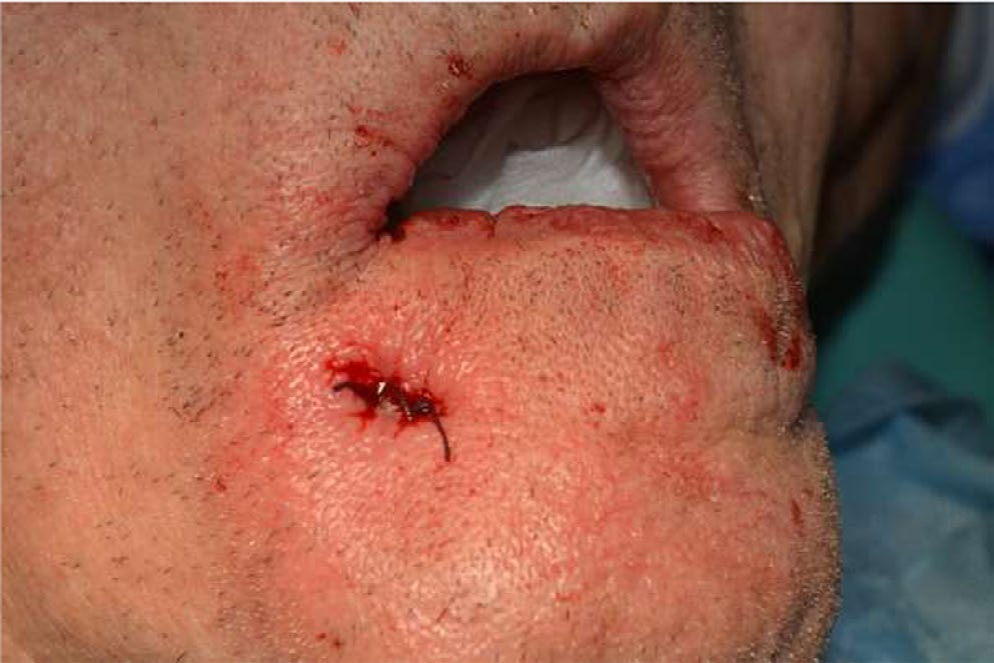
Extraoral view on operative region after suturing

In the next step, an incision was made at the ridge of the alveolar part of the mandible with scalpel blade No. 15, and the mucoperiosteal flap was reflected with Molt 9 periosteal elevator (Figure 4). The bone around the implant was drilled with rose bur on fast handpiece, and the implant was removed (Figure 5). Subsequently, using previous incision and flap designing, hyperplastic soft tissues were excised. Postoperative surgical wounds were dressed with simple interrupted resorbable sutures (Novosyn 2/0) (Figures 6 and 7).

The patient regained consciousness in the recovery room and was discharged home after reevaluation of his condition by the anesthesiologist. Postoperative antibiotic (300 mg of clindamycin every 8 h) and anti‐inflammatory medicine (250 mg of phenylbutazone every 12 h) were prescribed.

Follow‐up examination was performed the day after operation. Proper healing of the wound was observed, with mild edema of operative region. Sutures were left for spontaneous dissolution.

The excised soft tissues were sent for histopathological examination, which revealed hyperplastic epithelium and fibrous connective tissue with moderate infiltration of inflammatory cells. This description confirmed our initial diagnosis of epulis fissuratum.

## 3 DISCUSSION

The quality of life of the elderly is closely related to the condition of their bodies, general health, and psychosocial well‐being. Taking into account the various aspects in which oral health influences efficient nutrition and ensures facial aesthetics, it is also of great importance for their mental condition to ensure rehabilitation of stomatognathic system. Patients suffering from mental disorders, such as senile dementia, are always a therapeutic challenge for doctors, which results from the specificity of this disease. Logical contact with such patients is often difficult and sometimes even impossible, which excludes obtaining appropriate communication in the doctor‐patient relationship.^2^ Self‐care is definitely a huge problem for people with senile dementia, especially when the disease is constantly developing.^3^ The obvious consequence of these circumstances is, firstly, deterioration of oral hygiene and, subsequently, deterioration of the condition of their oral cavities.^10^ However, behavioral disorders may prevent cooperation when dental intervention is attempted.^11^ Moreover, their older age, frailty, mobility difficulties, and coexisting systemic diseases may limit treatment options.^12^ Taking all these factors into account, special attention should be paid to oral health prophylaxis in order to avoid difficult and complicated treatment.

A dental implant is one of the possibilities to replace missing teeth. Its use in the treatment of partially or completely edentulous patients has become an integral part of dental treatment nevertheless patients with mental illnesses face many difficulties in relation to implant treatment.^13^, ^14^ Treatment of such patients is usually possible only under general anesthesia in highly specialized centers with appropriate equipment and experienced medical staff. The issue of high costs of implant treatment, limited access to detailed information on the advancement of the disease, and little data on the effectiveness of treatment in patients with mental illnesses often lead to the resignation of this method in favor of removable acrylic dentures.^14^ It does not change the fact that the use of a prosthesis based on implants is associated with greater comfort expressed in its stability and retention, masticatory efficiency, and oral health‐related quality of life.^14^, ^15^

Implant‐supported overdentures are a predictable treatment option for completely edentulous mandibles. In this protocol, two dental implants to support the mandibular overdenture are considered to provide required conditions.^7^ As with any invasive medical procedure, periodic post‐treatment follow‐up is required to achieve the desired therapeutic effect. Negligence in this area can lead to serious complications, as discussed in this article, in the form of gradual tilting of the implant, loss of prosthesis retention, and the formation of fibrous mucosal hyperplasia. Chronic trauma and irritation by ill‐fitting denture are the two main etiological factors responsible for epulis fissuratum formation. Clinically, it presents as a mucogingival hyperplasia in the form of folds with a smooth surface with normal or erythematous overlying mucosa. Due to chronic irritation, it may be traumatized and get an ulcerated surface. Despite the fact that the epulis fissuratum may interfere with speaking and using the prosthesis, it may also undergo neoplastic transformation resulting from constant irritation.^8^ Therefore, after its surgical removal, histopathological examination is necessary.

The surgical methods of treatment include using any of the following: the conventional scalpel, electrocauterization, soft tissue lasers (carbon dioxide), and liquid nitrogen cryosurgery. The scalpel is an instrument that has been used in surgery from time immemorial due to its easy accessibility and low costs. Its main disadvantage is the need to control bleeding and place sutures after surgery. Electrocauterization is also used in oral surgery to remove epulis fissuratum with advantages of hemorrhage control and postoperative healing.^9^ The advantages of carbon dioxide laser noted were its tissue protective technique, asepticity, minimal postoperative pain and edema, rapid wound healing, insignificant scarring, and minimal functional impairment in the oral cavity.^9^, ^16^, ^17^ A recent study has shown that the liquid nitrogen cryosurgery has results equal to that of the carbon dioxide laser in terms of hemorrhage control and postoperative healing.^9^, ^18^

The choice of the method of treatment rests with the surgeon and should depend on his experience, skills, and hospital capabilities.

## 4 CONCLUSIONS

Dental treatment of patients with mental disorders is a challenge due not only to the difficulty of communication, but also to the problem of conducting follow‐up visits. Such patients are often not able to take care of their own needs, and the people taking care of them do not always pay attention to these needs. In the interest of treating the underlying disease, dental problems are often overlooked until they reveal themselves with visible symptoms. The aim of this article was to draw attention to the necessity of periodic dental examinations in mentally ill patients who cannot communicate with others because hidden ailments from the oral cavity can also significantly reduce their quality of life.

## CONFLICT OF INTEREST

None declared.

## AUTHOR CONTRIBUTIONS

DC: designed the study, collected the data, analyzed the data, and prepared the manuscript. MS: interpreted the data and involved in literature analysis. GT: collected data. JB‐G: involved in literature search. AJ‐N: planned the study, collected funds, and prepared the manuscript.

## ETHICAL APPROVAL

We obtained the necessary consent for this article.

## Data Availability

The references used to support the findings of this case report are listed in References.

## References

[1] World Health Organization International Classification of Disease. https://icd.who.int/browse11/l‐m/en#/http%3a%2f%2fid.who.int%2ficd%2fentity% 2f546689346. Accessed April 30, 2021

[2] Gao SS , Chu CH , Young FYF . Oral health and care for elderly people with Alzheimer's disease. Int J Environ Res Public Health. 2020;17(16):5713. 10.3390/ijerph17165713 PMC7460333 32784777

[3] Gao SS , Chen KJ , Duangthip D , Lo ECM , Chu CH . The oral health status of Chinese elderly people with and without dementia: a cross‐sectional study. Int J Environ Res Public Health. 2020;17(6):1913. 10.3390/ijerph17061913 PMC7143847 32183484

[4] Gil‐Montoya JA , Sánchez‐Lara I , Carnero‐Pardo C , et al. Oral hygiene in the elderly with different degrees of cognitive impairment and dementia. J Am Geriatr Soc. 2017;65:642‐647. 28024093 10.1111/jgs.14697

[5] Choi YS , Kim H , Rhee SH , et al. Multiple implant therapy with multiple inductions of general anesthesia in non‐compliant patients with schizophrenia: a case report. J Dent Anesth Pain Med. 2019;19(4):239‐244. 31501783 10.17245/jdapm.2019.19.4.239 PMC6726886

[6] Kern J‐S , Kern T , Wolfart S , Heussen N . A systematic review and meta‐analysis of removable and fixed implant‐supported prostheses in edentulous jaws: post‐loading implant loss. Clin Oral Impl Res. 2016;27:174‐195. 10.1111/clr.12531 PMC5024059 25664612

[7] Al‐Harbi FA . Mandibular implant‐supported overdentures: prosthetic overview. Saudi J Med Med Sci. 2018;6:2‐7. 30787808 10.4103/sjmms.sjmms_101_17 PMC6196685

[8] Mohan RPS , Verma S , Singh U , Agarwal N . Epulis fissuratum: consequence of ill‐fitting prosthesis. BMJ Case Rep. 2013;2013:bcr2013200054. 10.1136/bcr-2013-200054 PMC3736607 23867882

[9] Vyasarayani P , Madhumietha A , Gundlapalle P . Management of geriatric patient with epulis fissuratum using liquid nitrogen cryosurgery: a case report. J Indian Prosthodont Soc. 2014;14(1):115‐119. 24605008 10.1007/s13191-012-0156-3 PMC3935050

[10] Delwel S , Binnekade TT , Perez RSGM , Hertogh CMPM , Scherder EJA , Lobbezoo F . Oral hygiene and oral health in older people with dementia: a comprehensive review with focus on oral soft tissues. Clin Oral Investig. 2018;22:93‐108. 10.1007/s00784-017-2264-2 PMC5748411 29143189

[11] Scully C , Ettinger RL . The influence of systemic diseases on oral health care in older adults. J Am Dent Assoc. 2007;138:7‐14. 10.14219/jada.archive.2007.0359 17761840

[12] Henry RG , Smith BJ . Managing older patients who have neurologic disease: Alzheimer disease and cerebrovascular accident. Dent Clin North Am. 2009;53:269‐294. 19269397 10.1016/j.cden.2008.12.011

[13] Gupta R , Gupta N , Weber KK . Dental Implants. Treasure Island: StatPearls Publishing; 2020. 29262027

[14] Kim IH , Kuk TS , Park SY , Choi YS , Kim HJ , Seo KS . Prognosis following dental implant treatment under general anesthesia in patients with special needs. J Dent Anesth Pain Med. 2017;17(3):205‐213. 29090251 10.17245/jdapm.2017.17.3.205 PMC5647824

[15] Cardoso RG , Melo LA , Barbosa GA , et al. Impact of mandibular conventional denture and overdenture on quality of life and masticatory efficiency. Braz Oral Res. 2016;30(1):e102. 27737356 10.1590/1807-3107BOR-2016.vol30.0102

[16] Monteiro LS , Mouzinho J , Azevedo A , Câmara MI , Martins MA , La Fuente JM . Treatment of epulis fissuratum with carbon dioxide laser in a patient with antithrombotic medication. Braz Dent J. 2012;23(1):77‐81. 22460320 10.1590/s0103-64402012000100014

[17] de Arruda Paes‐Junior TJ , Cavalcanti SC , Nascimento DF , et al. CO(2) laser surgery and prosthetic management for the treatment of epulis fissuratum. ISRN Dent. 2011;2011:282361. 21991461 10.5402/2011/282361 PMC3170081

[18] Prohaska J , Jan AH . Cryotherapy. Treasure Island: StatPearls Publishing; 2020. 29493944

